# Case Report of a solitary benign spindle cell tumor in the deep thigh

**DOI:** 10.3389/fsurg.2026.1737867

**Published:** 2026-01-30

**Authors:** Haokang Zhang, Xinhua Yang, Guishi Li

**Affiliations:** Yantai Yuhuangding Hospital, The Affiliated Hospital of Qingdao University, Yantai, Shandong, China

**Keywords:** case report, sciatic nerve, spindle cell tumor, surgical excision, thigh

## Abstract

**Objective:**

This study aims to investigate the clinicopathological characteristics, imaging features, diagnostic approaches, and treatment strategies of spindle cell tumors of the thigh.

**Methods:**

We performed a retrospective analysis of the clinical data of a patient admitted to the Department of Orthopedics at Yantai Yuhuangding Hospital in 2025. The study period encompassed the preoperative assessment, surgical intervention, and a 10-month postoperative follow-up.

**Results:**

A 61-year-old male patient was admitted to our hospital with a 1 year history of right thigh pain without an obvious cause, accompanied by restricted mobility. Physical examination revealed a mass in the mid-posterior region of the right thigh with indistinct borders and skin numbness. Tenderness and percussion pain were noted in the right thigh, with pain limiting flexion and extension. Internal rotation was preserved, whereas external rotation was restricted. The Lasegue sign was positive. MRI examination showed a soft tissue mass located posteromedial to the proximal-to-mid segment of the right femur, raising suspicion for a tumor. The mass was irregular and lobulated with indistinct borders, presenting irregular, slightly prolonged signals on T1-weighted images and mixed high-/low-intensity signals on T2-weighted images. Intraoperatively, the tumor was located in the semimembranosus and semitendinosus muscles, spreading medially toward the anterior thigh. The tumor exhibited indistinct borders, lacked a capsule, and compressed the sciatic nerve. The tumor measured approximately 10 cm×5.6 cm×23 cm. Postoperative histopathological examination confirmed the diagnosis of a spindle cell tumor (occupying lesion of the root of the right thigh). The patient's postoperative pain and numbness were significantly alleviated.

**Conclusion:**

Solitary spindle cell tumors arising in the deep soft tissues of the thigh are clinically uncommon and often lack features related to neurofibromatosis type I (NF1). MRI is an important preoperative diagnostic modality. Complete surgical excision remains the treatment of choice and offers a good prognosis.

## 1 Introduction

Spindle cell tumor is a general term for tumors histologically composed of spindle-shaped cells. These tumors may originate from various tissues, including nerve, muscle, and fibrous tissue (1). Their clinical manifestations are heterogeneous; they may occur as solitary lesions or as part of the multiple manifestations of neurofibromatosis type I (NF1) (1, 2). Solitary spindle cell tumors most commonly present in the skin. However, tumors originating from deep soft tissues and invading major nerve trunks of the thigh (such as the sciatic nerve), in the absence of features related to NF1, are relatively uncommon. In this article, we report a case of a spindle cell tumor involving the sciatic nerve admitted to our department. B reviewing the related literature, we analyzed its diagnostic and therapeutic aspects with the aim of improving our understanding of this disease.

## 2 Case report

### 2.1 Clinical data

The patient was a 61-year-old man Who presented with a chief complaint of right thigh pain and numbness for approximately 1 year, accompanied by restricted mobility. He had previously been treated for lumbar disc herniation at an outside hospital, receiving oral ibuprofen, acupuncture, and physical therapy, all of which were ineffective. His symptoms progressively worsened over the last 2 months, with the development of mild skin numbness. The patient had a history of gastric cancer surgery. Physical examination revealed tenderness in the right thigh, and deep palpation identified a firm mass with indistinct borders. No café-au-lait spots, axillary freckling, and iris hamartomas were observed. The patient denied any family history of NF1; however, but both his father and brother had died from leukemia.

### 2.2 Specialized examination and palpation

**Figure 1:**
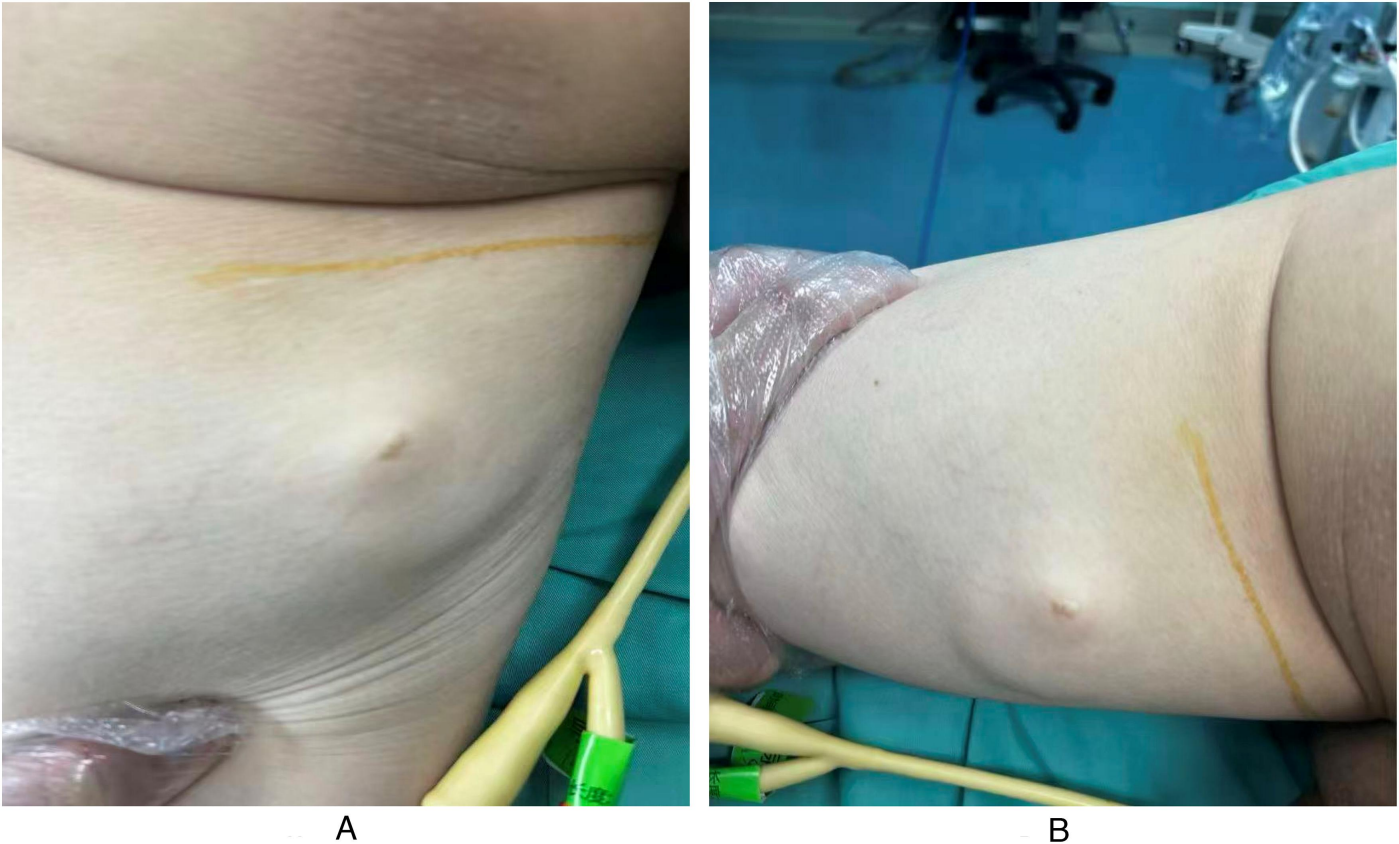
**(A)** Preoperative manifestations of the mass on the body surface. **(B)** Postoperative manifestations of the mass on the body surface.

A deep-seated mass was palpable in the middle segment of the posterior right thigh. The mass had a moderately firm texture, indistinct borders, slight longitudinal mobility along the course of the nerve trunk, and poor transverse mobility. Sensory abnormalities of the right lower limb included numbness over the anterolateral aspect of the lower leg, dorsum of the foot, and first toe; decreased sensation over the posterior aspect of the lower leg, lateral border of the foot, and sole; and mild dysesthesia involving the posterior thigh and posterior lower leg. Tapping over the projection of the swelling elicited a radiating numbness to the dorsum of the foot (positive Tinel sign). Manual muscle testing (MMT) was performed to assess motor function, with results showing grade 4/5 strength in ankle dorsiflexion, ankle plantarflexion, and great toe dorsiflexion. The Lasegue sign was positive, Patrick’s test and reverse Patrick’s test yielded mixed positive and negative findings. Preoperative manifestations of the mass on the body surface are shown in Figures 1A,B.

### 2.3 Examination results upon admission

As detailed in Table 1.

**Table 1:** Results of routine laboratory examinations upon admission.

Category	Parameter	Result
Urinalysis	Protein	Negative (−)
	Glucose	Negative (−)
	Occult blood	Negative (−)
	Leukocytes	Negative (−)
	Urobilinogen	Positive (+)
	pH	7.5
	Specific gravity	1.012
Hematology	White blood cell (WBC) count	7,000/mm^3^
	Hemoglobin (Hb)	8.5 g/dL
	Hematocrit (Hct)	29.2%
	Platelet count	36.5×10^3^/mm^3^
	Neutrophils (%)	58.9%
Markers	C-reactive protein (CRP)	<3.13 mg/L
	Rheumatoid factor (RF)	<10.0 IU/mL
	Carcinoembryonic antigen (CEA)	2.44 ng/mL
	Alpha-fetoprotein (AFP)	3.91 ng/mL
	Cytokeratin 19 fragment (CYFRA 21-1)	3.49 ng/mL
	Carbohydrate antigen 19-9 (CA 19-9)	10.5 U/mL

### 2.4 Imaging examinations

**Figure 2:**
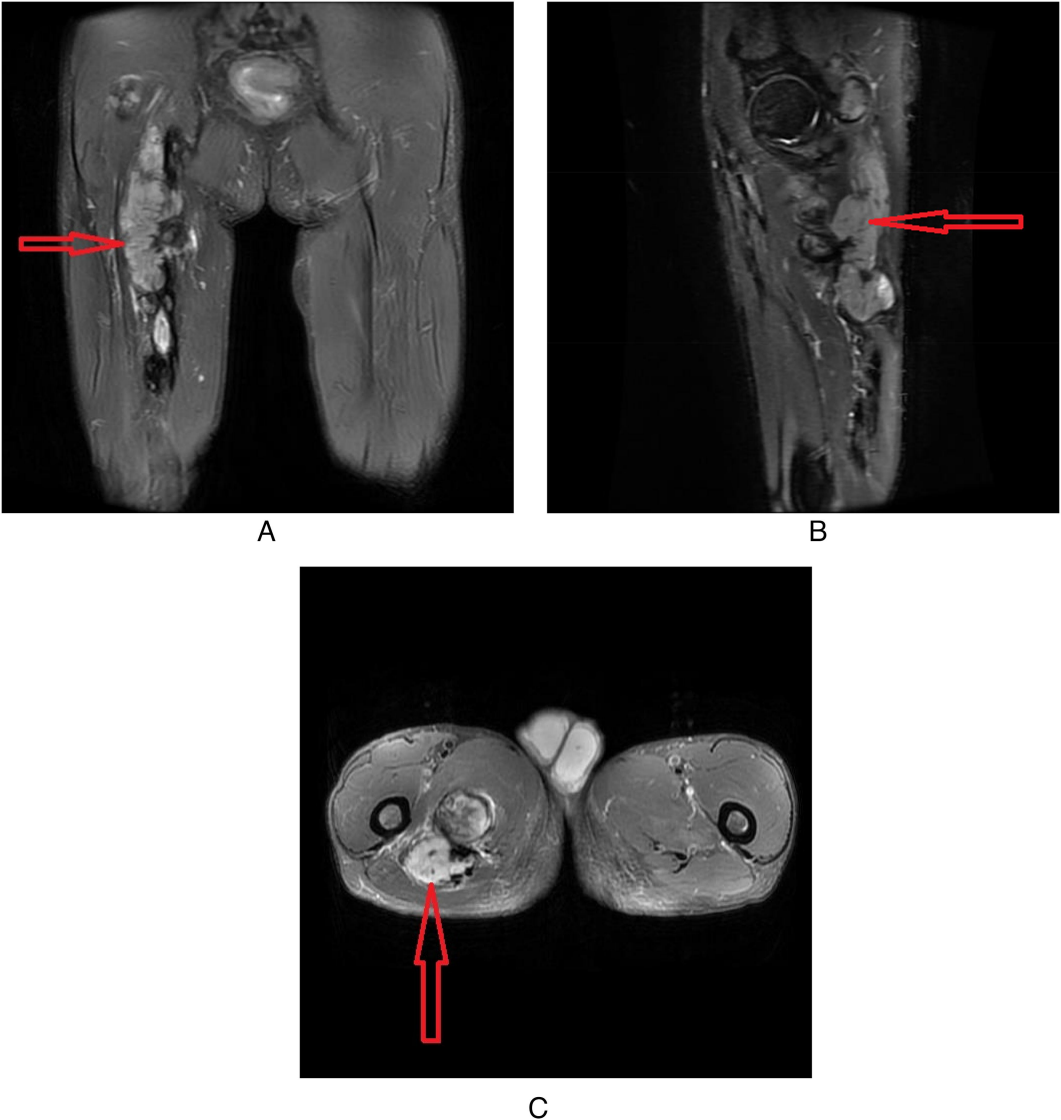
**(A)** MRI features of the tumor in the coronal plane. **(B)** MRI features of the tumor in the sagittal plane. **(C)** MRI features of the tumor in the axial plane.

Magnetic resonance imaging (MRI) revealed a mass with indistinct borders along the course of the sciatic nerve in the posterosuperior segment of the right thigh. The soft tissue mass was located posteromedial to the proximal-to-mid segment of the right femur and was suggestive of a tumor (Figures 2A–C). The mass was irregular and lobulated with indistinct borders, measuring approximately 8.7 cm×4.3 cm×21.2 cm, without significant signal change. The mass demonstrated slightly prolonged signals on T1-weighted images and mixed high-/low-intensity signals on T2-weighted images.

### 2.5 Treatment and results

**Figure 3:**
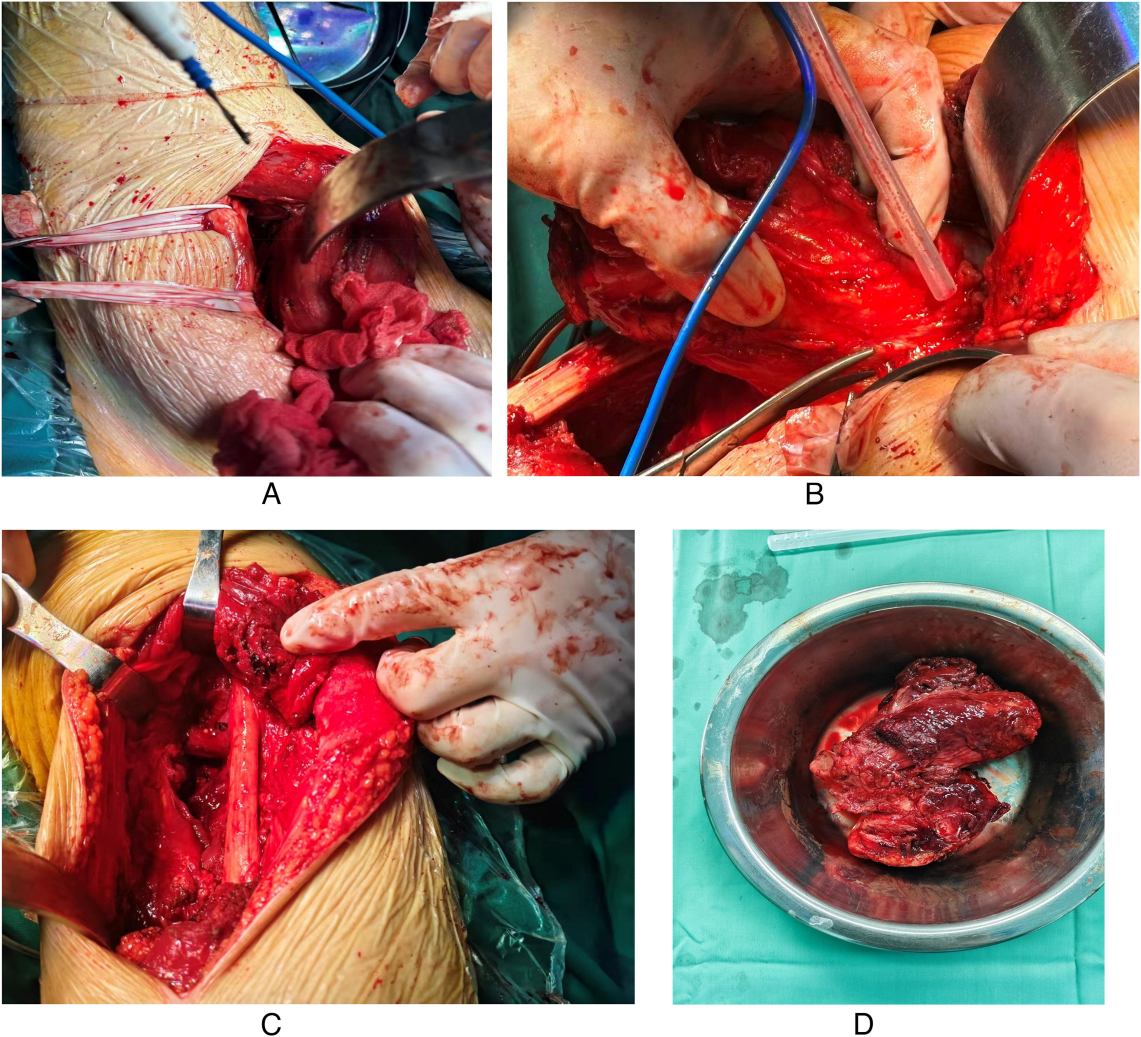
**(A)** Selection of the posterior approach and tumor exposure. **(B)** Tumor dissection and resection. **(C)** Exploration of the sciatic nerve. **(D)** Gross specimen description.

After admission, preoperative preparation was completed. The patient underwent excision of the right femoral mass under general anesthesia. The incision was made along the posterior thigh. A figure-of-eight skin incision was made over the mass, extending approximately 18 cm in length (Figure 3A). The skin, subcutaneous tissue, and deep fascia were incised to expose the intermuscular space. The tumor was located within the semimembranosus and semitendinosus muscles and extended medially toward the anterior thigh, with ill-defined margins. The lesion lacked a capsule and compressed the sciatic nerve (Figure 3B). The sciatic nerve was carefully retracted and protected (Figure 3C). Proximal exploration revealed normal muscle and soft tissue encapsulating the tumor. The tumor measured approximately 10 cm×5.6 cm×23 cm and was irregular in shape. It contained multiple tumor nodules and lacked a capsule. The extent of tumor invasion was identified by dissecting the tumor at the attachment of the gluteus maximus. The muscle was reflected to define the exact tumor margins. The tumor was completely removed by cutting the involved muscle attachments at the pelvic insertion point, outside the compartment. The specimen was sent for histopathological examination (Figure 3D). Postoperatively, the patient reported significant improvement in pain, compression, and numbness compared to the preoperative state. Intraoperative photographs are displayed in the figures.

### 2.6 Gross specimen

**Figure 4:**
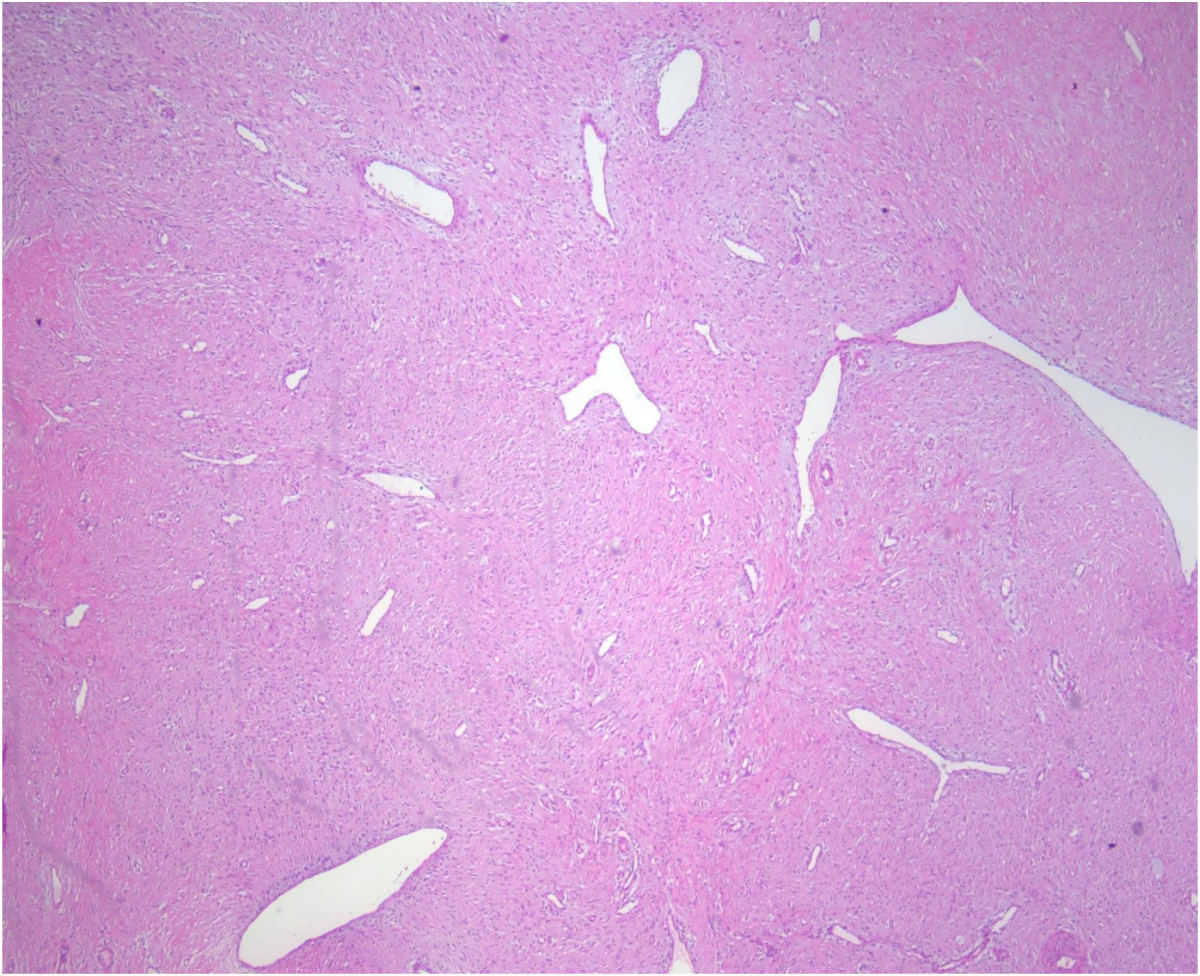
H&E staining (100× magnification).

The gross specimen was a dark red, spindle-shaped mass, without a capsule, measuring 10 cm×5.6 cm×23 cm. The cut surface was hard and displayed a whorled pattern. On microscopic examination (H&E staining), the mass was composed of spindle cells arranged in a relatively dense, phyllodes-like pattern. Fibrous structures were seen in the stroma. The tumor cells were relatively uniform (Figure 4).

#### 2.6.1 Pathological diagnosis

(Right thigh soft tissue mass) spindle cell tumor: Based on the combined histolomorphological findings and immunohistochemical (IHC) profile, the lesion is consistent with a fibroblastic/myofibroblastic proliferative neoplasm, showing features that support the diagnosis of benign fibromatosis.

Surgical margin: No tumor infiltration was identified at the muscle excision margins.

Immunohistochemistry (IHC) profile: The immunophenotype of the spindle cell population was characterized based on the results in Table 2.

**Table 2:** Summary of the immunohistochemistry (IHC) profile.

Marker	Expression	Significance
S-100	Strongly positive (Strongly +)	Characteristic marker for Schwannian differentiation
Vimentin (Vim)	Positive (+)	Consistent with mesenchymal origin
INI-1	Positive (+)	INI-1 protein expression is retained
Ki-67	Low (\sim1–2%)	Indicates low proliferative activity (consistent with benignity)
CD34	Focally positive (focally +)	Contributes to differential diagnosis (e.g., neurofibroma, spindle cell lipoma)
β-catenin	Negative (−)	Rules out aggressive fibromatosis
SMA	Negative (−)	Rules out myofibroblastic differentiation
Desmin	Negative (−)	Rules out myogenic tumors
CD31, EMA, MUC4, SS18-SSX, STAT6	All negative (−)	Excludes specific sarcoma subtypes and mimics

### 2.7 Postoperative follow-up

Postoperatively, the patient's radiating pain and numbness in the right thigh were significantly relieved compared to the preoperative state. The patient's motor strength recovered to grade V. At 1-, 3-, 6-, and 10-month follow-up visits after surgery, Tinel's sign was negative. Functional recovery of the affected limb was satisfactory. Follow-up MR, showed no evidence of tumor recurrence.

## 3 Discussion

This study reports the case of a solitary spindle cell tumor originating from the deep soft tissues of the thigh in a 61-year-old male patient.

### 3.1 Similarities to previous research

First, in terms of clinical features, previous studies have shown that deep-seated solitary spindle cell tumors mainly occur in young and middle-aged adults, with no obvious gender predilection (3). Clinical symptoms are closely related to the location and size of the tumor and mainly reflect progressive nerve compression, presenting as local pain, distal numbness, sensory disorder, and muscle weakness. These findings are consistent with the chronic pain, sensory abnormalities, and limited mobility presented by the patient in this case, which reflect the typical clinical manifestations of such tumors (2, 4).

Second, regarding imaging diagnosis, MRI is widely recognized as the preferred preoperative method for examining spindle cell tumors (5). Most studies report that such tumors often appear as fusiform masses along the course of the nerve trunk, demonstrating isointense or slightly hypointense signals on T1-weighted images (T1WIs) and hyperintense signals on T2-weighted images (T2WIs). Although the tumor in this case did not show the typical “target sign,” its location along the sciatic nerve course, ill-defined borders, and signal characteristics are consistent with the imaging features of most neurogenic spindle cell tumors reported in the literature. These findings support the value of MRI for preoperative localization and qualitative assessment (6–8).

Third, in terms of pathology and immunophenotype, previous studies have shown that spindle cell tumors are histologically composed of spindle-shaped cells arranged within a myxoid or collagenous stroma. Immunohistochemical positivity for vimentin supports a mesenchymal origin, and a low Ki-67 proliferation index indicates benign biological behavior (9). The pathological findings in this case, including spindle cells arranged in a phyllodes-like pattern, a fibrous stroma, vimentin positivity, and a Ki-67 proliferation index of only 1%–2%, are highly consistent with the characteristics of benign spindle cell tumors reported in previous studies (10).

Finally, in terms of treatment and prognosis, most studies agree that complete surgical resection is the first-line treatment for symptomatic or progressively enlarging solitary spindle cell tumors. Patients with benign histopathology and negative surgical margins have a good prognosis and a low recurrence rate (11). The successful surgical resection and favorable 10-month postoperative follow-up results in this patient further verify these findings (12).

### 3.2 Differences from previous research

First, in terms of disease incidence and origin, most previous studies have reported that solitary spindle cell tumors more commonly arise in the skin or superficial soft tissues, and some cases are associated with NF1 or present as multiple lesions. In contrast, the tumor in this case is a sporadic solitary lesion without NF1-related manifestations, originating from the deep semimembranosus and semitendinosus muscles of the thigh and directly compressing the sciatic nerve (8, 13). Such deep-seated, nerve-compressing, NF1-unrelated solitary spindle cell tumors are clinically rare and differ from the more common origins and associated backgrounds of spindle cell tumors reported in the literature.

Second, in terms of imaging manifestations, the “target sign” (a low signal center with a high-signal periphery on T2WI) is considered a suggestive imaging feature of some neurogenic spindle cell tumors (such as neurofibromas). However, in this case, the tumor showed irregular, slightly prolonged signals on T1-weighted images and mixed high-/low-intensity signals on T2-weighted images without the typical “target sign,” which is consistent with the imaging performance of a small number of atypical spindle cell tumors reported in the literature but differs from the typical manifestations described in most studies. Such atypical imaging findings increase the difficulty of preoperative differential diagnosis and require careful distinction from schwannoma, aggressive fibromatosis, and other lesions (6, 7).

Third, in terms of immunophenotype, previous studies have shown that some neurogenic spindle cell tumors (such as schwannomas) often exhibit specific immunophenotypic characteristics, such as S-100 positivity, in combination with certain myogenic marker expressions. In this case, the immunophenotype was characterized by strong S-100 positivity (suggesting Schwann cell differentiation), focal CD34 positivity, and negative β-catenin, SMA, and desmin, thereby excluding aggressive fibromatosis and myogenic tumors. This immunophenotypic profile differs slightly from those reported for some neurogenic spindle cell tumors in previous studies and enriches the immunophenotypic data on sporadic, deep-seated benign spindle cell tumors.

In conclusion, this case supplements the clinical, imaging, and pathological data on rare, sporadic, deep-seated solitary spindle cell tumors of the thigh. Its consistency with previous studies confirms the universality of the clinical characteristics, diagnostic methods, and treatment principles for benign spindle cell tumors, while the differences remind clinicians to pay attention to atypical manifestations in clinical practice, improve the accuracy of preoperative diagnosis through comprehensive analysis of multiple examinations, and ensure the effectiveness of surgical treatment and a good prognosis for patients.

## 4 Conclusion

In summary, this case adds to the clinical, imaging, and pathological data on a rare, sporadic, deep-seated spindle cell tumor of the thigh. Its consistency with previous studies confirms the universality of the clinical characteristics, diagnostic methods, and treatment principles for benign spindle cell tumors, while the discrepancies remind clinicians to pay attention to atypical manifestations in practice, improve the accuracy of preoperative diagnosis through comprehensive analysis of multiple examinations, and ensure the effectiveness of surgical treatment and a favorable prognosis for patients.

## Data Availability

The original contributions presented in the study are included in the article/Supplementary Material; further inquiries can be directed to the corresponding author.
